# Ultrasound and Contrast-Enhanced Ultrasound Characteristics Associated With cN1 and Microscopic pN1 in Papillary Thyroid Carcinoma

**DOI:** 10.3389/fendo.2021.810630

**Published:** 2022-01-24

**Authors:** Wen Li, Shusheng Qiu, Ling Ren, Qiuyang Li, Shaowei Xue, Jie Li, Yan Zhang, Yukun Luo

**Affiliations:** ^1^ Department of Ultrasound, Medical School of Chinese People’s Liberation Army (PLA), Beijing, China; ^2^ Department of Ultrasound, The First Medical Center, Chinese PLA General Hospital, Beijing, China; ^3^ Department of Surgery, ZiBo Central Hospital, Zibo, China; ^4^ Department of Pathology, The First Medical Center, Chinese PLA General Hospital, Beijing, China

**Keywords:** ultrasound, contrast-enhanced ultrasound, papillary thyroid carcinoma, lymph node metastasis, cN1, microscopic pN1

## Abstract

**Objectives:**

Lymph node metastases (LNMs) could be stratified into clinical N1 (cN1) and microscopic pN1 (pathological N1), which bear different biological behavior and prognosis. Our study aimed to investigate the associations between LNMs and primary tumor’s US (ultrasound) and CEUS (contrast-enhanced ultrasound) characteristics based on the stratification of LNMs into cN1 and microscopic pN1 in papillary thyroid carcinoma (PTC).

**Methods:**

From August 2019 to May 2020, 444 consecutive PTC patients who underwent preoperative neck US and CEUS evaluation were included. According to regional lymph node status, the patients were classified into cN1 group versus cN0 (clinical N0) group and microscopic pN1 group versus pN0 (pathological N0) group. For multiple PTCs, the largest one was selected for the evaluation of US, CEUS and clinical features. Univariate and multivariate analyses were performed to determine independent predictors of cN1 and microscopic pN1.

**Results:**

85 cN1 versus 359 cN0 patients and 117 microscopic pN1 versus 242 pN0 patients were analyzed. Multivariate logistic regression analysis showed that <55-years-old (OR: 2.56 (1.08–6.04), male [OR: 2.18 (1.22–3.91)], large size [OR: 2.59 (1.71–3.92)], calcification [OR: 3.88 (1.58–9.51)], and hyper-enhancement [OR: 2.78 (1.22–6.30)] were independent risk factors of cN1, while <55-years-old [OR: 1.91 (1.04–3.51)], large size [OR: 1.56 (1.003–2.42)], multifocality [OR: 1.67 (1.04–2.66)] were independent risk factors of microscopic pN1.

**Conclusions:**

For patients with PTC, young age, male, large size, calcification, and hyper-enhancement were independent predictors of cN1, while young age, large size and multifocality were independent predictors of microscopic pN1.

## Introduction

Papillary thyroid carcinoma (PTC) is the most common thyroid carcinoma, comprising more than 90% of all thyroid cancers (1). Most PTC patients have good prognosis. However, cervical lymph node metastases (LNMs) are common in PTCs and could increase the recurrence risk and mortality outcomes of PTCs (2, 3). Central compartment dissection has been performed for the PTC patients prone to LNMs (4), yet it might increase the likelihood of morbidity, including hypocalcemia and is unnecessary for those with low-risk LNMs. In addition, postoperative radioiodine therapy might be considered for aggressive PTCs (5). Therefore, it is essential to evaluate the risk factors of LNMs and establish a procedure to screen preoperatively for aggressive PTCs, thereby assisting with the therapeutic planning.

Although ultrasound is the preferred imaging modality for the assessment of LNMs in PTC patients (4), preceding studies (3, 6) found that conventional ultrasound missed 33–90% of LNMs in PTC patients. Instead of direct detection, investigators focused on the sonographic features of PTCs to predict LNMs, and contrast-enhanced ultrasound (CEUS) has also been utilized to screen for aggressive PTCs from indolent ones and predict LNMs.

Some investigations (7–13) have explored the PTC’s US and CEUS features associated with LNMs and confirmed their values in predicting LNMs. However, LNMs could be stratified into clinical N1 (cN1, observed on US) and microscopic pN1 (pathological N1), which might bear different biological behavior and prognostic values. cN1 PTCs are deemed to be more aggressive and have worse prognosis than microscopic pN1 PTCs (1, 4, 14–17). Very similar recurrence risks and mortality outcomes could be seen in patients with pN0 and microscopic pN1 (1). The differences in biological behavior and prognosis between cN1 and microscopic pN1 might signify that the characteristics of US and CEUS of primary tumor could also be different.

Hence, the present study aimed to investigate the associations between LNMs and each of the US and CEUS characteristics of PTCs based on the stratification of LNMs into cN1 and microscopic pN1.

## Materials And Methods

### Patients

This retrospective study was conducted from August 2019 to May 2020. The inclusion criteria were as follows: 1. Patients who underwent open surgery (initial therapy); 2. The definitive pathological diagnosis was PTC; 3. Both conventional US and CEUS examinations were carried out within one month before operation. The following were excluded: 1. Central lymph node (level VI) dissection was not performed; 2. Patients with other subtypes of thyroid carcinomas. Thus, a total of 444 patients (331 females and 113 males; mean age, 43.50 ± 11.08 years; range, 18-69 years) were identified and enrolled. For multifocal PTCs, the largest one was selected. Ultimately, 444 PTC lesions (median size, 1.0 (0.7,1.5) cm) were chosen for this study.

This study was approved by Medical Ethics Committee of Chinese PLA General Hospital (S2019-178-02) and the written informed consents were waived because the patients’ data were assessed retrospectively and anonymously.

### Stratification of LNMs

LNMs include cN1 and microscopic pN1. cN1 is defined as clinically detectable macroscopic LNMs which could be observed on US. Microscopic pN1 could only be detected by histopathologic review. For the cN1 unconfirmed by preoperative cytology, they should be validated by postoperative pathology in our study. For this purpose, we matched the suspicious LNMs observed on US to the corresponding lymph nodes on pathology based on location and size. As long as any of them was demonstrated by postoperative pathology, the cN1 would be established in our study.

According to the regional lymph node status, the patients were classified into cN1 group versus cN0 group [including microscopic pN1 and pN0 (pathological N0)] and microscopic pN1 group versus pN0 group.

### Conventional US and CEUS Examinations

This investigation was carried out with machines from four vendors: a Mindray Resona7 (Shenzhen, China) equipped with a L14-5WU transducer for conventional US and a L11-3U for CEUS, a Philips iU22 (Bothell, WA, USA) equipped with a L12-5 transducer for conventional US and a L9-4 for CEUS, an ACUSON Sequoia (Siemens, Germany) equipped with a 18L6 for conventional US and a 10L4 for CEUS as well as an ACUSON S2000 (Siemens, Germany) equipped with a 9L4 transducer for conventional US and CEUS. Patients were scanned in supine position with neck fully exposed. By grayscale ultrasound, the size, position, composition, echogenicity, shape, margin, calcification and halo of the nodule were thoroughly examined. The regional lymph nodes were also meticulously scrutinized.

The largest plane of the tumor was chosen and US machine was then switched to CEUS mode. And the focus was always placed deeper than the examined nodule to diminish the microbubble disruption. The contrast medium was SonoVue (Bracco Imaging S.p.A, Milan, Italy), which was administered intravenously through the antecubital vein as a bolus at a dose of 1.8-2.0 mL followed by 5 mL of saline flush. The real-time microbubble perfusions within the nodules and surrounding tissues were observed for a minimum of 2 min and recorded in the hard drive of the system.

### Image Interpretation and Analysis

Two sonologists (Y.K.L. and Y.Z.) with more than 10 years of experience in thyroid ultrasonography, were blinded to the clinicopathological information and performed a retrospective review of the US and CEUS sonograms independently. Discrepancies in the interpretation were resolved by consensus.

Concerning the US features, the following were evaluated for each nodule: composition (solid or almost completely solid, others), echogenicity (hyperechoic or isoechoic, hypoechoic, very hypoechoic), shape (wider-than-tall, taller-than-wide), margin (smooth, lobulated or irregular), calcification (absent, present), halo (absent, present), suspicious extrathyroidal extension on US (absent, present). The following characteristics were used to evaluate the suspicious extrathyroidal extension on US: the nodule abuts the thyroid capsule, which displays the signs of interruption (loss of the echogenic capsule line); and the nodule interrupts the capsule and invades the surrounding tissues (18–20). With respect to the regional lymph nodes, according to the previous studies (21–26), US-based detection of highly suspicious LNM was based on the following features: 1) microcalcification; 2) cystic change; 3) hyper-echogenicity; 4) rounded shape with increased anteroposterior diameter; 5) peripheral vascularization; 6) irregular margin and loss of hilum. The suspicious lymph nodes on US were routinely recommended for fine needle biopsy in our institution.

The thyroid nodules were assessed on CEUS relative to the surrounding thyroid parenchyma. Compared to that of the surrounding thyroid tissue, the peak intensity of the nodule was classified into (1): hypo-enhancement (2), iso-enhancement (3), hyper-enhancement. According to the preceding studies (27, 28), the presence of tumor-adjacent hyper-enhancement area(s) was indicative of high invasiveness of tumors. This feature was defined as the hyper-enhancement area(s) abutting on tumor. The ring, band-like or patchy enhancement area(s) could be observed in this feature. As to the hyper-enhancement nodules, this feature presented the extension of enhancement region.

### Histological Analysis

All specimens were classified in accordance with the WHO Classification of Tumors of Endocrine Organs (4th edition) by experienced pathologists who were blinded to the medical history and sonographic findings. Pathological diagnosis was considered as the gold standard for the study.

### Statistical Analysis

The statistical software used in this study was SPSS, version 21.0 (SPSS Inc, Chicago, IL). *P* value of <0.05 was considered statistically significant. The patients were categorized as cN1 group versus cN0 group and microscopic pN1 group versus pN0 group. Continuous variables with normal distribution were summarized as mean ± standard deviation, otherwise they were presented as median (lower quartile, upper quartile). Categorical variables were summarized as frequencies and percentages. Continuous variables were assessed using the unpaired Student *t* test or Mann Whitney U test. Categorical variables were evaluated by Pearson’s chi-square or Fisher’s exact test. Variables, that were significant in the univariate analysis or were clinically relevant, were selected into a binary logistic regression model using an enter method to further identify the independent risk factors associated with cN1 and microscopic pN1 separately and establish the prediction models. Odds ratios (ORs) with 95% confidence intervals (CIs) were calculated. The receiver operating characteristic (ROC) curves obtained by MedCalc were used to determine the best cut-off values for the continuous variables (tumor size) and to assess the models’ diagnostic performance. The corresponding area under the ROC curve (AUC), sensitivity, and specificity were calculated at the optimal cut-off values.

## Results

### Histological Findings

444 PTCs included 5 aggressive variants (only tall cell variant) and 439 non-aggressive variants, including classical, follicular, oncocytic and warthin-like variants. LNMs were found in 202 patients (45.50%). Among them, 85 patients were found cN1 and 117 were found microscopic pN1. In 85 cN1 patients, at least one involved lymph node could be observed on US. 44 of 85 cN1 patients were confirmed by preoperative cytology and the rest were demonstrated by postoperative pathology based on the size and location. Extra-nodal extension occurred in 15 of 85 cN1 patients, which was documented in the operative report.

**Figure 1:**
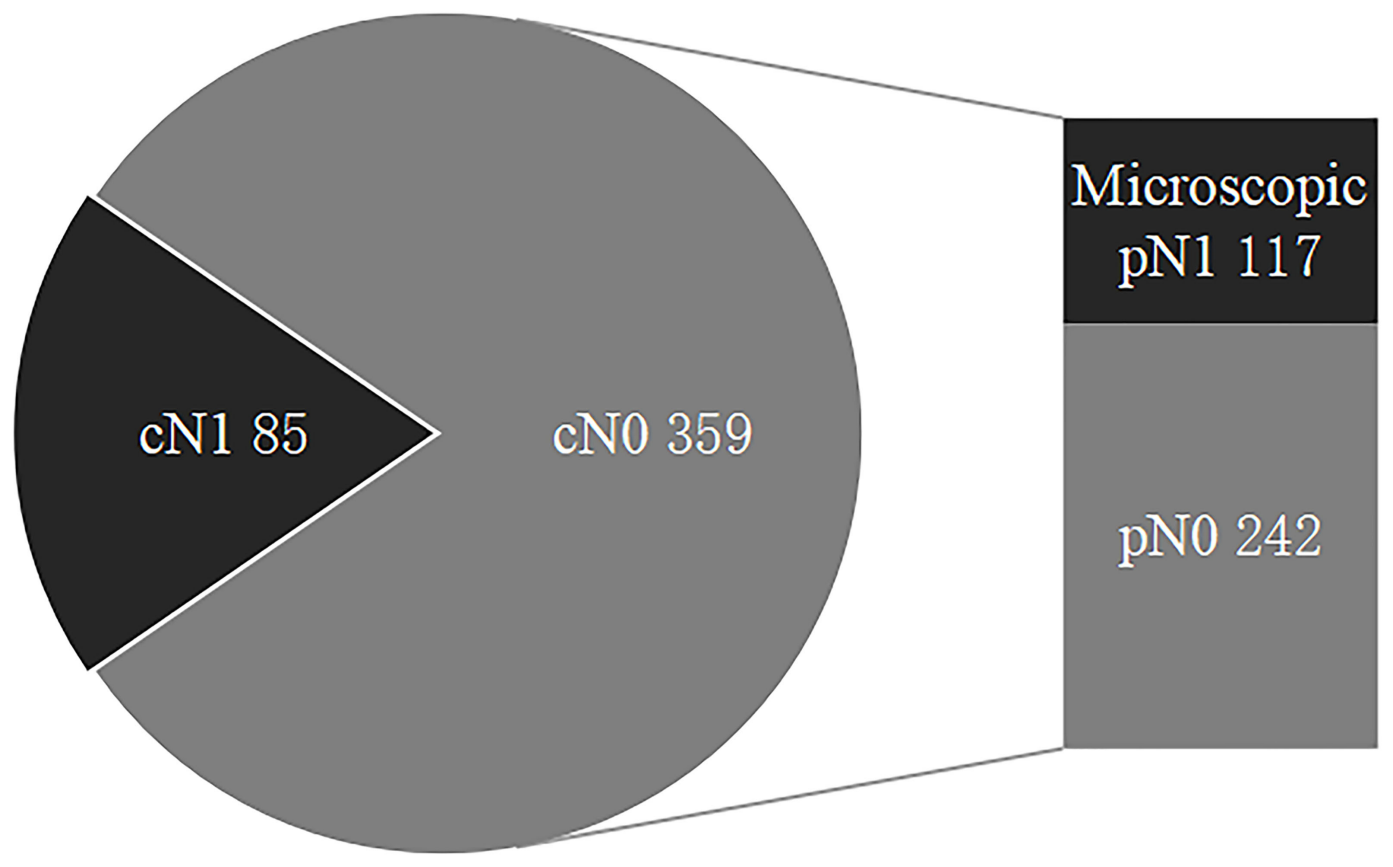
The status of regional lymph nodes in papillary thyroid carcinoma. LNMs include cN1 and microscopic pN1, and cN0 are comprised of microscopic pN1 and pN0. LNMs, lymph node metastases; cN1, clinical N1; Microscopic pN1, microscopic pathological N1; cN0, clinical N0; pN0, pathological N0.

Thus, 85 cN1 patients versus 359 cN0 patients (including 117 microscopic pN1 patients and 242 pN0 patients) and 117 microscopic pN1 patients versus 242 pN0 patients were analyzed in our study (**Figure 1**).

### Comparison of PTC’s US and CEUS Features Between cN1 Patients and cN0 Patients

The age, gender, size, multifocality, extrathyroidal extension on US, calcification and enhancement intensity differed significantly between patients with cN1 and cN0 (*p*<0.05) (**Table 1**). However, there were no significant differences in composition, echogenicity, shape, margin, halo as well as tumor-adjacent hyper-enhancement area(s) (*p*>0.05) (**Table 1**).

**Table 1:** Clinical and sonographic characteristics of cN1 PTCs and cN0 PTCs.

Characteristics	cN1 PTCs (n = 85)	cN0 PTCs (n=359)	*P* value
Age, y	38.71 ± 10.87	44.63 ± 10.84	<0.0001^*^
<55	77 (90.59)	284 (79.11)	0.015^*^
Gender			<0.0001^*^
Male	37 (43.53)	76 (21.17)	
Size (Maximum diameters, cm)	1.50 (1.00,2.45)	1.00 (0.60,1.20)	<0.0001^*^
Multifocality	51 (60.00)	152 (42.34)	0.003^*^
Suspicious extrathyroidal extension on US			<0.001^*^
Absent	14 (16.47)	130 (36.21)	
Present	71 (83.53)	229 (63.79)	
Composition			0.877
Solid or almost completely solid	83 (97.65)	354 (98.61)	
Others	2 (2.35)	5 (1.39)	
Echogenicity			0.083
Hyperechoic or isoechoic	2 (2.35)	1 (0.28)	
Hypoechoic	74 (87.06)	303 (84.40)	
Very hypoechoic	9 (10.59)	55 (15.32)	
Shape			0.311
Wider-than-tall	38 (44.71)	139 (38.72)	
Taller-than-wide	47 (55.29)	220 (61.28)	
Margin			0.935
Smooth	3 (3.53)	16 (4.46)	
Lobulated or irregular	82 (96.47)	343 (95.54)	
Presence of calcification	79 (92.94)	240 (66.85)	<0.0001^*^
Presence of halo	14 (16.47)	38 (10.58)	0.129
Enhancement intensity			<0.0001^*^
Hypo-enhancement	14 (16.47)	129 (35.93)	
Iso-enhancement	37 (43.53)	172 (47.91)	
Hyper-enhancement	34 (40.00)	58 (16.16)	
Presence of tumor-adjacent hyper-enhancement area (s)	18 (21.18)	91 (25.35)	0.422

cN1, clinical N1; cN0, clinical N0; PTC, papillary thyroid carcinoma.

Data were presented in mean ± standard deviation, median (lower quartile, upper quartile) and number (percent).

*These p values are of <0.05.

### Multivariate Logistic Regression Analysis for cN1

Multivariate logistic regression analysis was performed for all significant variables identified in the univariate analysis. Among them, size was added to the multivariate model as a continuous variable. Additionally, given that the PTCs with halo (encapsulated tumors) tend to present rich blood supply but highly favorable prognosis (29), this feature was also added to the multivariate model to check for possible confounding effects.

**Figure 2:**
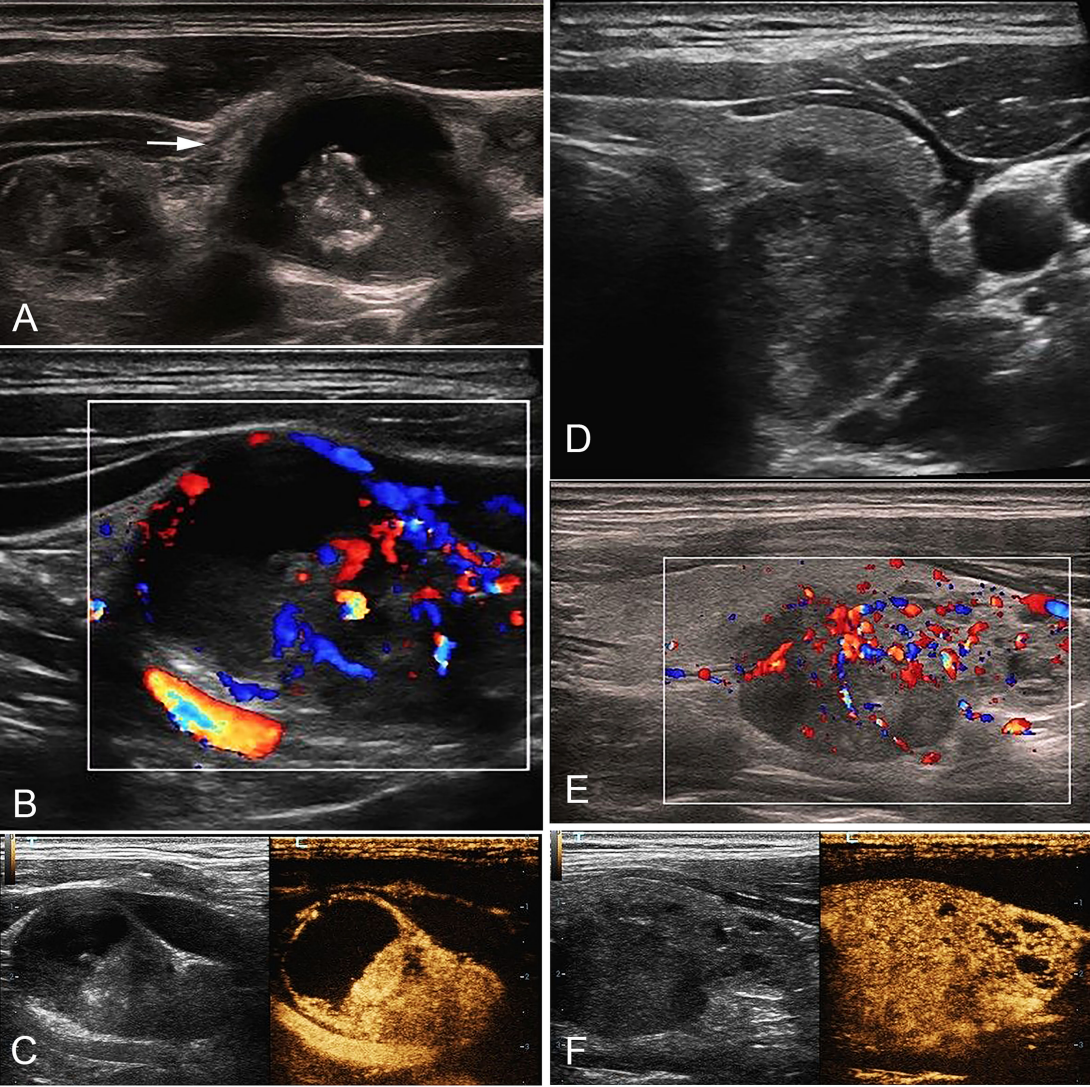
A 35-year-old male with cN1 PTC. **(A)** Sonogram of the largest metastatic lymph node (arrow) in level IV indicates irregular margin, loss of hilum, calcification, hyper-echogenicity, and cystic change in the node. **(B)** CDFI shows non-hilar vascularization. **(C)** CEUS indicates heterogeneous hyper-enhancement and perfusion defect in the node. **(D)** Sonogram of the PTC lesion shows a solid hypo-echogenic nodule with irregular margin and a taller than wide shape, measuring 1.2*1.4*1.9cm. **(E)** CDFI indicates abundant blood flow signals. **(F)** CEUS shows that the PTC lesion presents pervasive hyper-enhancement. PTC, papillary thyroid carcinoma; cN1, clinical N1; CDFI, color doppler flow imaging; CEUS, contrast-enhanced ultrasound.

The predictive factors for cN1 were <55-years-old (OR: 2.56 (1.08–6.04), *p*: 0.032), male (OR: 2.18 (1.22–3.91), *p*: 0.009), size (OR: 2.59 (1.71–3.92), *p*<0.0001), calcification (OR: 3.88 (1.58–9.51), *p*: 0.003), and hyper-enhancement (OR: 2.78 (1.22–6.30), *p*: 0.015) (**Figure 2**), whereas the others were dependent on cN1 (*p*>0.05). The AUC was 0.557 for age (95% CI: 0.510–0.604), 0.612 for gender (95% CI: 0.565–0.657), 0.749 for size (95% CI: 0.706–0.789), 0.630 for calcification (95% CI: 0.584–0.675), and 0.658 for enhancement intensity (95% CI: 0.612–0.702), respectively. The best cut-off value of tumor size was >1.4 cm.

**Figure 3:**
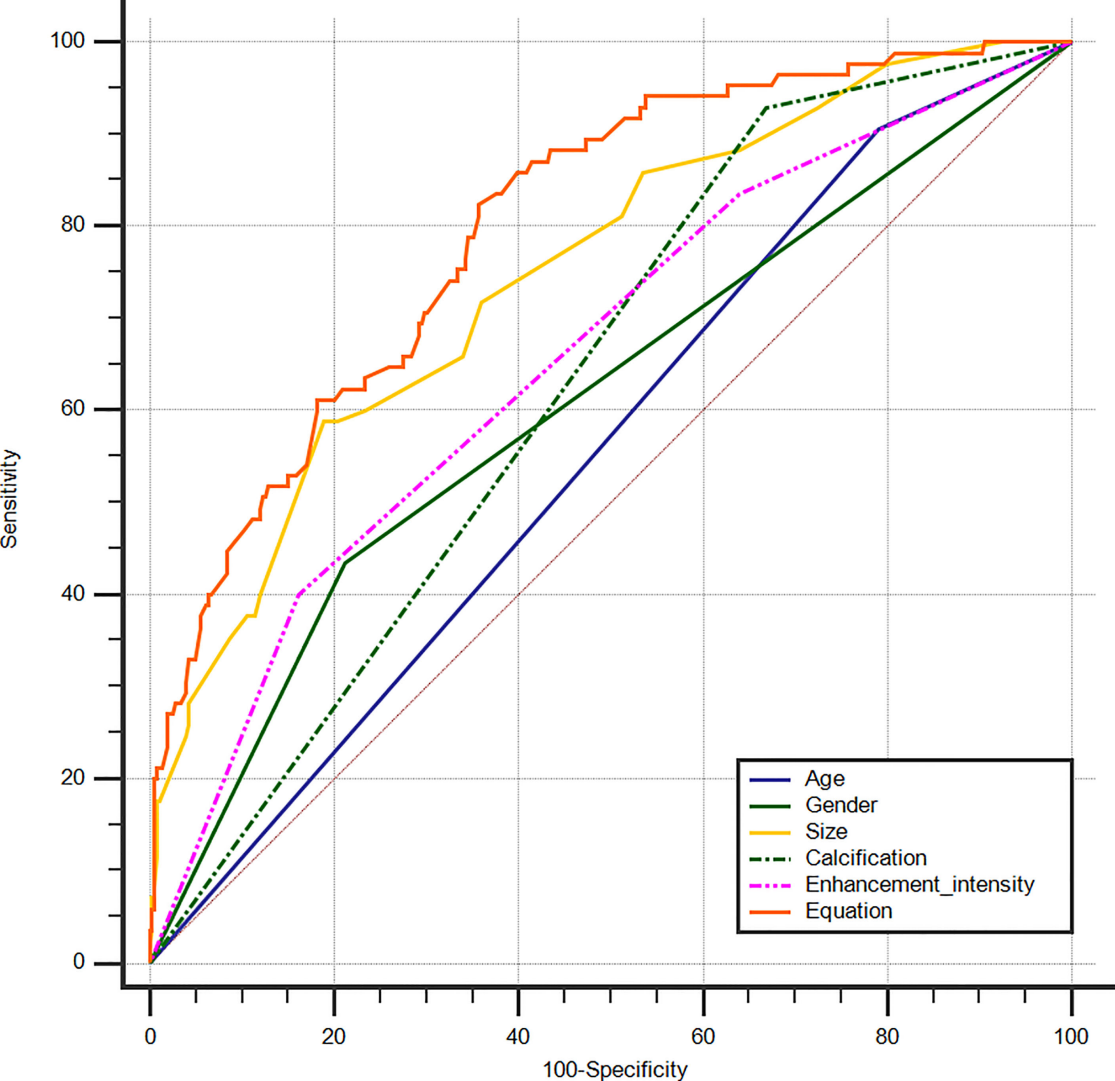
ROC curves of PTC age (AUC=0.557), gender (AUC=0.612), size (AUC=0.749), calcification (AUC=0.630), enhancement intensity on CEUS (AUC=0.658) and Equation (AUC=0.802) for the prediction of cN1. ROC, receiver operating characteristic; PTC, papillary thyroid carcinoma; AUC, area under the curve; CEUS, contrast-enhanced ultrasound; cN1, clinical N1.

An equation was established with the significant predictive factors: logit (*p*) = -5.289 + 0.902 × age + 0.736 × gender + 0.919 × size + 0.575 × iso-enhancement + 0.876 × hyper-enhancement. The AUC of 0.802 (95% CI: 0.762–0.838) for equation suggested a significant difference compared with the single factors (*p*<0.05 for all) (**Figure 3**). The calcification showed the highest sensitivity (92.94%) in predicting cN1, while enhancement intensity indicated the highest specificity (83.84%). The diagnostic accuracy of size was the highest (76.80%) (**Table 2**).

**Table 2:** ROC analysis for predicting cN1 in PTCs.

Characteristics	AUC	95% CI	Sensitivity	Specificity	Accuracy
Age	0.557	0.510–0.604	90.59%	20.89%	34.23%
Gender	0.612	0.565–0.657	43.53%	78.83%	72.07%
Size^*^	0.749	0.706–0.789	58.82%	81.06%	76.80%
Calcification	0.630	0.584–0.675	92.94%	33.15%	44.59%
Enhancement intensity	0.658	0.612–0.702	40.00%	83.84%	75.45%
Predictive equation	0.802	0.762–0.838	82.35%	64.35%	67.79%

ROC, receiver operating characteristic; cN1, clinical N1; PTC, papillary thyroid carcinoma; AUC, area under the curve; CI, confidence interval.

*The best cut-off value of tumor size was >1.4 cm.

### Comparison of PTC’s US and CEUS Features Between Microscopic pN1 Patients and pN0 Patients

Statistically significant differences were found in age, size, multifocality and calcification between microscopic pN1 patients and pN0 patients (*p*<0.05) (**Table 3**). But there were no significant differences in gender, extrathyroidal extension on US, composition, echogenicity, shape, margin, halo, enhancement intensity as well as tumor-adjacent hyper-enhancement area(s) (*p*>0.05) (**Table 3**).

**Table 3:** Clinical and sonographic characteristics of microscopic pN1 PTCs and pN0 PTCs.

Characteristics	Microscopic pN1 PTCs (n = 117)	pN0 PTCs (n = 242)	*P* value
Age, y	42.75 ± 11.11	45.54 ± 10.61	0.022^*^
<55	99 (84.62)	185 (76.45)	0.074
Gender			0.243
Male	29 (24.79)	47 (19.42)	
Size (Maximum diameters, cm)	1.00 (0.70,1.45)	0.80 (0.60,1.20)	0.002^*^
Multifocality	60 (51.28)	92 (38.02)	0.017^*^
Suspicious extrathyroidal extension on US			0.430
Absent	39 (33.33)	91 (37.60)	
Present	78 (66.67)	151 (62.40)	
Composition			1.000
Solid or almost completely solid	115 (98.29)	239 (98.76)	
Others	2 (1.71)	3 (1.24)	
Echogenicity			0.470
Hyperechoic or isoechoic	0 (0)	1 (0.41)	
Hypoechoic	103 (88.03)	200 (82.65)	
Very hypoechoic	14 (11.97)	41 (16.94)	
Shape			0.277
Wider-than-tall	50 (42.74)	89 (36.78)	
Taller-than-wide	67 (57.26)	153 (63.22)	
Margin			0.907
Smooth	5 (4.27)	11 (4.55)	
Lobulated or irregular	112 (95.73)	231 (95.45)	
Presence of calcification	88 (75.21)	152 (62.81)	0.019^*^
Presence of halo	11 (9.40)	27 (11.16)	0.612
Enhancement intensity			0.264
Hypo-enhancement	48 (41.03)	81 (33.47)	
Iso-enhancement	49 (41.88)	123 (50.83)	
Hyper-enhancement	20 (17.09)	38 (15.70)	
Presence of tumor-adjacent hyper-enhancement area (s)	28 (23.93)	63 (26.03)	0.668

Microscopic pN1, microscopic pathological N1; pN0, pathological N0; PTC, papillary thyroid carcinoma.

Data were presented in mean ± standard deviation, median (lower quartile-upper quartile) and number (percent).

*These p values are of <0.05.

### Multivariate Logistic Regression Analysis for Microscopic pN1

To control more confounders, the variables added to the multivariate analysis were identical to those added to the multivariate analysis of cN1. The predictive factors for microscopic pN1 were <55-years-old (OR: 1.91 (1.04–3.51), *p*: 0.036), size (OR: 1.56 (1.003–2.42), *p*: 0.049), multifocality (OR: 1.67 (1.04–2.66), *p*: 0.033). The AUC was 0.541 for age (95% CI: 0.488–0.593), 0.601 for size (95% CI: 0.548–0.652), and 0.566 for multifocality (95% CI: 0.513–0.618), respectively. The best cut-off value of tumor size was >1.0 cm.

**Figure 4:**
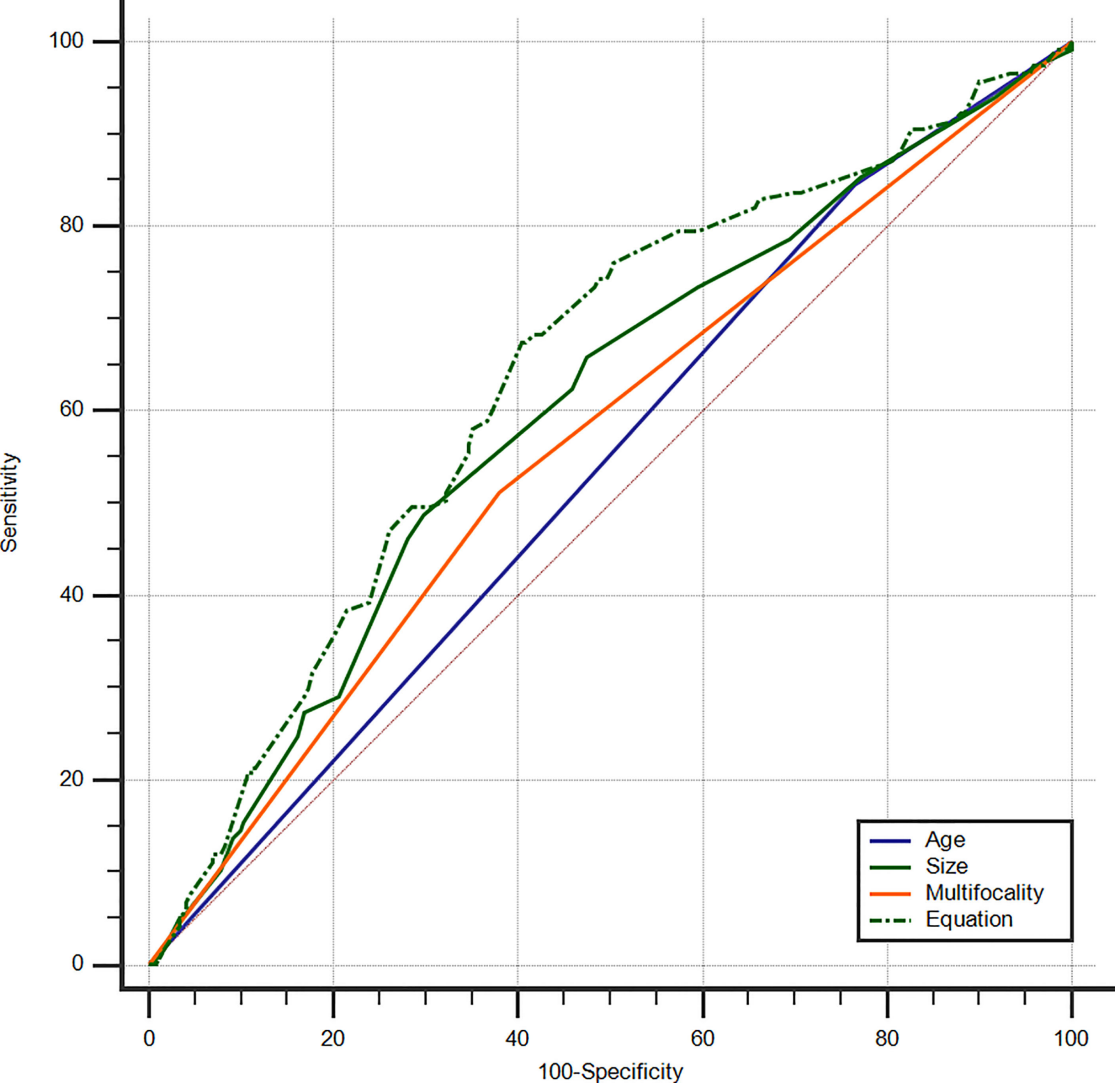
ROC curves of PTC age (AUC=0.541), size (AUC=0.601), multifocality (AUC=0.566), and Equation (AUC=0.639) for the prediction of microscopic pN1. ROC, receiver operating characteristic; PTC, papillary thyroid carcinoma; AUC, area under the curve; Microscopic pN1, microscopic pathological N1.

An equation was also created using the significant predictive factors: logit (*p*) = -1.853 + 0.609 × age + 0.391 × size + 0.502 × multifocality. The AUC of the predictive equation was 0.639 (95% CI: 0.586–0.688), higher than the single factors (*p*<0.05 for age and multifocality, but not for size) (**Figure 4**). The age had the highest sensitivity (84.62%) in predicting microscopic pN1, whereas the size indicated the highest specificity (70.25%). The size also achieved the highest accuracy (63.23%) (**Table 4**).

**Table 4:** ROC analysis for predicting microscopic pN1 in PTCs.

	AUC	95% CI	Sensitivity	Specificity	Accuracy
Age	0.541	0.488–0.593	84.62%	23.55%	43.45%
Size^*^	0.601	0.548–0.652	48.72%	70.25%	63.23%
Multifocality	0.566	0.513–0.618	51.28%	61.98%	58.50%
Predictive equation	0.639	0.586–0.688	67.52%	59.50%	62.12%

ROC, receiver operating characteristic; Microscopic pN1, microscopic pathological N1; PTC, papillary thyroid carcinoma; AUC, area under the curve; CI, confidence interval.

*The best cut-off value of tumor size was >1.0 cm.

## Discussion

LNMs can be classified into cN1 and microscopic pN1, which might vary in biological behavior and prognosis. cN1 is defined as clinically detectable macroscopic LNMs which could be observed on US. Microscopic pN1 could only be found by histopathology. Microscopic pN1 carries a smaller recurrence risk than cN1 but possesses similar recurrence risks and mortality outcomes with pN0 (1, 4, 14–17). Microscopic pN1 might have little impact on the risk of structural recurrence and disease specific survival (1). Consequently, it could be hypothesized that primary tumor’s US and CEUS features associated with cN1 and microscopic pN1 might differ. Our study explored the connections between LNMs and primary tumor’s US and CEUS characteristics based on the stratification of LNMs into cN1 and microscopic pN1 and confirmed this hypothesis.

We discovered that independent risk factors for cN1 were <55-years-old, male, large size, calcification and hyper-enhancement, while those for microscopic pN1 were <55-years-old, large size and multifocality. CEUS, a pure blood pool imaging technique, could be used to assess the blood perfusion of tumors (30). As the angiogenesis is essential in the occurrence, development, invasion, and metastasis of PTCs, it could be speculated that PTCs with hyper-enhancement (rich blood supply) possessed high invasiveness and an increased probability of LNMs, which could further develop into clinically detectable LNMs (cN1). In our study, hyper-enhancement on CEUS was correlated with cN1 rather than with microscopic pN1. The similar conclusion was drawn by other studies (7, 10) that hyper-enhancement on CEUS suggested a high risk of LNMs. In these studies, the LNMs included both cN1 and microscopic pN1. It has been reported that calcification was an independent predictor of LNMs for patients with PTC (31–33). In our study, calcification was associated with cN1, but not with microscopic pN1. Male is associated with a worse prognosis, constituting a non-independent adverse prognostic factor (34, 35). Some studies (36, 37) have reported that male was an independent predictor of LNMs in patients with PTC. In our study, male was connected with cN1 rather than microscopic pN1. Size is an independent prognostic factor of PTCs and has been demonstrated to be associated with LNMs by many studies (7–9, 11, 12). This conclusion was validated in our study. The optimal cut-off value of size for the prediction of cN1 was 1.4cm, whereas that for the prediction of microscopic pN1 was 1.0cm. Previous studies (31, 32, 37) found that young age was connected with LNMs. This conclusion was demonstrated in our study, although Jianming Li et al. (36) reported that old age was a risk factor of LNMs. Multifocality has been reported to be associated with an increased risk of LNMs (8, 32, 37, 38) and it was identified to be an independent risk factor of microscopic pN1 in our study.

The eighth edition of the American Joint Committee on Cancer (AJCC) Manual mentions that the current challenge for clinical staging is that the conventional US cannot confidently classify the lymph nodes as clinically negative lymph nodes (cN0) or clinically positive lymph nodes (cN1) before Surgery (1). According to our study, for PTCs with the predictive factors of cN1, very careful scrutiny of neck ultrasound was needed to screen for the potential macroscopic LNMs. If suspicious or indeterminate lymph nodes were found, lymphatic or intravenous CEUS should be recommended (21, 22, 39), thereby increasing the confidence in diagnosis and biopsy, and improving the detection rate of LNMs, especially for junior sonologists. For cN0 patients with PTC, the predictive factors (young age, large size and multifocality) were suggestive of an increased probability of microscopic LNMs. It might be better that prophylactic central compartment dissection was performed for patients with these factors.

A more demolitive surgery is accompanied by a risk of increased postoperative complications, such as neck hematoma, hypoparathyroidism, and recurrent laryngeal nerve injury (40–42). Therefore, it is of great significance to accurately identify LNMs before surgery, which could impact on the extent of surgery. Our findings could serve as preoperative supplementary markers for determining the status of LNMs, thereby enhancing the accuracy and detection rate of LNMs and assisting the surgeons in determining the optimal extent of surgery.

Our study had some limitations. First, this is a retrospective, single-center study. Second, our institution is a tertiary referral hospital. Additionally, many patients with papillary thyroid microcarcinoma were subjected to active surveillance or thermal ablation therapy rather than surgery. Therefore, surgery patients in our hospital might have the higher staging and sample selection bias might exist. A multi-center study was needed to solve these problems. Third, our study cohort lacked postoperative follow-up data. Future studies should investigate postoperative follow-up sonography in our institution and compare recurrence and survival rates.

In conclusion, PTCs with the risk factors, including <55-years-old, male, large size, calcification and hyper-enhancement, have an increased probability of cN1. Cervical lymph nodes should be carefully scrutinized. For cN0 patients with PTC, young age, large size and multifocality were associated with an increased likelihood of microscopic pN1. Prophylactic central compartment dissection might be appropriate for these patients.

## Data Availability Statement

The raw data supporting the conclusions of this article will be made available by the authors, without undue reservation.

## Ethics Statement

The studies involving human participants were reviewed and approved by Medical Ethics Committee of Chinese PLA General Hospital. The ethics committee waived the requirement of written informed consent for participation. Written informed consent was obtained from the individual(s) for the publication of any potentially identifiable images or data included in this article.

## Author Contributions

The conception and design of the study: WL, SQ, YZ, and YL. Acquisition of data: WL and SQ. Analysis and interpretation of data: LR, QL, SX, and JL. Drafting the article: WL and SQ. The article and final approval of the version to be submitted: YZ and YL.

## Funding

This work was supported by grants (81901746 to YZ) from National Natural Science Foundation of China, grants (81771834 to YL) from National Natural Science Foundation of China, and grants (2019MBD-040 to YL) from Chinese PLA General Hospital.

## Conflict of Interest

The authors declare that the research was conducted in the absence of any commercial or financial relationships that could be construed as a potential conflict of interest.

## Publisher’s Note

All claims expressed in this article are solely those of the authors and do not necessarily represent those of their affiliated organizations, or those of the publisher, the editors and the reviewers. Any product that may be evaluated in this article, or claim that may be made by its manufacturer, is not guaranteed or endorsed by the publisher.
